# Evaluating an in-home multicomponent cognitive behavioural programme to manage concerns about falls and associated activity avoidance in frail community-dwelling older people: Design of a randomised control trial [NCT01358032]

**DOI:** 10.1186/1472-6963-11-228

**Published:** 2011-09-20

**Authors:** Tanja AC Dorresteijn, GA Rixt Zijlstra, Kim Delbaere, Erik van Rossum, Johan WS Vlaeyen, Gertrudis IJM Kempen

**Affiliations:** 1 CAPHRI School for Public Health and Primary Care, Faculty of Health, Medicine and Life Sciences, Department of Health Services Research, Maastricht University, Maastricht, The Netherlands; 2 Falls and Balance Research Group, Neuroscience Research Australia and University of New South Wales, Randwick, Sydney, Australia; 3 Centre of Research on Autonomy and Participation and Centre of Research on Technology in Care, Zuyd University of Applied Sciences, Heerlen, The Netherlands; 4 Clinical Psychological Science, Maastricht University, Maastricht, The Netherlands; 5 Department of Psychology, University of Leuven, Leuven, Belgium

## Abstract

**Background:**

Concerns about falls are frequently reported by older people. These concerns can have serious consequences such as an increased risk of falls and the subsequent avoidance of activities. Previous studies have shown the effectiveness of a multicomponent group programme to reduce concerns about falls. However, owing to health problems older people may not be able to attend a group programme. Therefore, we adapted the group approach to an individual in-home programme.

**Methods/Design:**

A two-group randomised controlled trial has been developed to evaluate the in-home multicomponent cognitive behavioural programme to manage concerns about falls and associated activity avoidance in frail older people living in the community. Persons were eligible for study if they were 70 years of age or over, perceived their general health as fair or poor, had at least some concerns about falls and associated avoidance of activity. After screening for eligibility in a random sample of older people, eligible persons received a baseline assessment and were subsequently allocated to the intervention or control group. Persons assigned to the intervention group were invited to participate in the programme, while those assigned to the control group received care as usual. The programme consists of seven sessions, comprising three home visits and four telephone contacts. The sessions are aimed at instilling adaptive and realistic views about falls, as well as increasing activity and safe behaviour. An effect evaluation, a process evaluation and an economic evaluation are conducted. Follow-up measurements for the effect evaluation are carried out 5 and 12 months after the baseline measurement. The primary outcomes of the effect evaluation are concerns about falls and avoidance of activity as a result of these concerns. Other outcomes are disability and falls. The process evaluation measures: the population characteristics reached; protocol adherence by facilitators; protocol adherence by participants (engagement in exposure and homework); opinions about the programme of participants and facilitators; perceived benefits and achievements; and experienced barriers. The economic evaluation examines the impact on health-care utilisation, as well as related costs.

**Discussion:**

A total number of 389 participants is included in the study. Final results are expected in 2012.

**Trial registration:**

NCT01358032

## Background

Falls and concerns related to falls are very common in community-dwelling older people. About one in three older people living in the community experiences at least one fall each year, of which roughly half results in an injury [1,2]. Interestingly, around two-thirds of older people report concerns related to falls [3], of which roughly half report activity avoidance as a result of these concerns [4-8]. Concerns about falls can be present in both people who have fallen and people who have not [9]. These concerns have been linked to decreased balance performance, decreased mobility, functional decline, low quality of life, institutionalisation and falls [3,10-13]. Recent research has shown that concerns about falls can lead to falls irrespective of any physiological fall risk [13].

Concerns about falls can be considered as a multifactorial problem [14,15]. As a consequence, successful programmes should not only target concerns about falls but should also focus on aspects like increasing self-efficacy and a sense of control regarding the risks of falling, setting realistic goals for increasing activity, changing the environment to reduce the fall risk and promoting physical activity to increase strength and balance [15-19].

Cognitive behavioural therapy could be seen as a suitable strategy to reduce concerns about falls by modifying patterns of thoughts (cognition) and actions (behaviour) that contribute to the concern.

One of the programmes with proven effectiveness is the Dutch version of 'A Matter of Balance' (AMB-NL) [20]. This multicomponent cognitive behavioural group programme consists of eight weekly group sessions and a booster session after six months. It has shown favourable effects on concerns about falls, perceived control over falling and daily activity, after at least 8 months of follow-up. In addition, significantly fewer recurrent fallers were observed in this group after 14 months of follow-up [15]. Notwithstanding these positive outcomes, approximately 40% of the participants attended less than five sessions out of a total eight, mainly because of health problems [21]. It seems that, in particular, frail older people refrained from attending the group programme. This is also seen in other group programmes that explicitly address concerns about falls, and target frail older people living in the community [18,22].

The aim of this project is to develop an in-home programme to enable frail older people to participate, as well as people who prefer an in-home approach rather than a group approach [Dorresteijn, Zijlstra, Van Eijs, Vlaeyen, Kempen: Older people's preferences regarding programme formats for managing concerns about falls, submitted]. This paper presents the design of a randomised controlled trial evaluating 'A Matter of Balance at Home' (AMB-Home) in frail older people, living in the community in the Netherlands. The objectives of this trial are to conduct: (1) an effect evaluation to determine the effects of this in-home programme on concerns about falls and fall-related activity avoidance, and additional outcomes including disability and fall incidents; (2) a process evaluation to determine the feasibility of the programme; and (3) an economic evaluation uncovering the impact of the programme on health-care utilisation and related costs.

## Methods/Design

### Design

**Figure 1:**
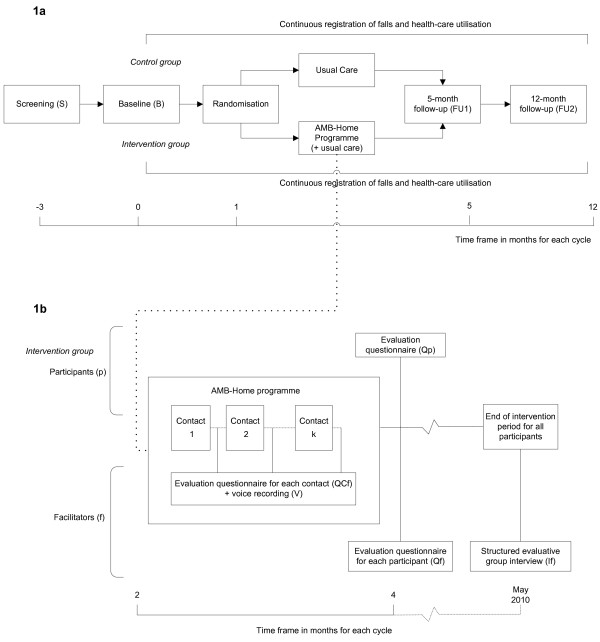
**Study design**. **1a** Displaying effect and economic evaluation. S = screening; B = baseline; FU1 = 5-month follow-up; FU2 = 12-month follow-up. **1b** Displaying process evaluation. QCf = questionnaire filled in by facilitator for each participant for each contact, V = voice recording for selection of contacts, Qf = questionnaire filled in by facilitator for each participant, Qp = questionnaire filled in by participant after programme, and If = group interview with facilitators.

The study concerns a two-group randomised controlled trial with a baseline measurement and follow-up measurements after 5 (directly after the programme) and 12 months (see Figure 1). The selection of potential participants was performed between March and December 2009 in four consecutive cycles. Each cycle lasted about 15 months and included: screening for eligible participants; baseline measurement; stratified randomisation; the intervention period; and follow-up measurements. The Medical Ethics Committee of the Maastricht University/Academic Hospital Maastricht in The Netherlands approved this trial.

### Recruitment of participants

Three communities, Maastricht, Sittard-Geleen, and Heerlen, situated in the southeast of The Netherlands have been selected for participation in the trial. The municipal registry offices selected 11,490 addresses of community-dwelling people of 70 years or over in their district, at random. To screen for eligibility, people received a short postal questionnaire with a freepost envelope, as well as information about the trial and an informed consent form. After a fortnight, reminder letters were sent. The questionnaire assessed socio-demographics and fall-related variables and inclusion and exclusion criteria.

Older people were included if they met all of the following criteria: 1) they reported at least some concerns about falls; 2) they reported at least some associated avoidance of activity; 3) they perceived their general health as fair or poor [23]; 4) they lived in the community; 5) they were 70 years of age or older; and 6) they were willing to participate (signed informed consent form). People were excluded if they were confined to bed, were restricted by the permanent use of a wheelchair, were waiting for a nursing home admission, experienced substantial hearing or vision impairments or they failed the shortened version of the Abbreviated Mental Test (AMT4) [24] assesses cognitive impairment and, subsequently, the Telephone Interview Cognitive Status (TICS) [25]. In addition, a restriction was applied to couples; to prevent reciprocal influencing only one partner of a couple was allowed to participate in the trial. Lots were drawn if this selection process was necessary.

### Randomisation

Participants were assigned to the programme or the no-treatment control group directly after the baseline measurement using stratified randomisation. This randomisation was performed in blocks of two on the basis of one prognostic factor: the level of concern about falls (some, regular, often, and very often). Computerised alternative allocation was used in the randomisation process, which was performed by an external agency.

### Programme

#### Development of the programme

AMB-Home is based on the Dutch version of a cognitive behavioural group programme for older people living in the community named 'A Matter of Balance' (AMB-NL). AMB-NL is a translated, adapted and evaluated version of 'A Matter of Balance' [20], a programme that originated in the USA [18]. In the development process of the home-based version for frail older people, all components and techniques of the group programme were assessed on their appropriateness for the new targeted population, the in-home setting, and future implementation in Dutch health-care. Therefore, experts (n = 8, see Acknowledgements) in the domain of programme development and behavioural change were consulted and a pilot study with six volunteers was conducted to test the programme initial feasibility.

Substantial elements of the AMB-NL programme, such as cognitive restructuring, a DVD for modelling purposes, i.e. presenting stories of other people reporting concerns about falls and their solutions, and the discussions on themes related to fall prevention themes are maintained in AMB-Home. However, several adaptations have been made. First, the programme of AMB-Home consists of seven sessions (three home visits and four telephone contacts) in contrast to the eight sessions and a booster session after six months in AMB-NL. Second, the formulation and review of personal action plans, regarding the themes of the sessions, receive a more prominent role throughout the programme to encourage active participation [21,26]. Third, the physical exercises performed in AMB-NL are not included in AMB-Home since supervision of the appropriate execution of these exercises is not feasible. Fourth, a new component is added to home visit 3. During this visit, the participant actually practises a specific (fear-related) problem in daily life in the presence of the programme facilitator. Lastly, motivational interviewing is added as a discussion technique, to motivate participants to change their beliefs and behaviour, regarding concerns about falls and activity avoidance. See Table 1 for additional information on the similarities and differences of both programmes.

**Table 1:** Main Topics of the In-home Multicomponent Cognitive Behavioural Intervention

Session 1: Concerns about falls (home visit; 60 min)
Introduction of the programme
Discuss theme of the session using a DVD:
Beliefs and disbeliefs about concerns about falls Thoughts related to concerns about falls and their effect on feelings and behaviour
**Session 2: Exploring thoughts and concerns about falls (home visit; 60 min)**
Review previous session and homework*
Discuss theme of the session using a checklist:
Thoughts and attitudes related to concerns about falls and challenging them Adaptive responses to counter misconceptions about falls Unhelpful thoughts and their effect on feelings and behaviour
Identifying activities in which concerns about falls are experienced
Formulating an action plan to perform an activity safely in which concerns about falls are experienced*

**Session 3: Physical exercise (telephone; 35 min)**
Review previous session and homework*
Discuss theme of the session using a checklist:
Misconceptions regarding physical exercise for older people Potential consequences of inactivity and benefits of physical activity Staying or becoming physically active to prevent falls Recognising and overcoming barriers to staying or becoming physically active
Formulating an action plan to perform a physical exercise activity in daily life*

**Session 4: Asserting oneself (telephone; 35 min)**
Review previous session and homework*
Discuss theme of the session using a checklist and a leaflet about fall hazards:
Association between assertiveness and fall prevention Potential barriers and benefits of being assertive Reducing concerns about falls and falls-risks by being assertive Recognising potential environmental fall hazards in one's home and community
Formulating an action plan to be assertive related to fall prevention*

**Session 5: Overcoming personal barriers (home visit; 75 min)**
Review previous session and homework*
Recognising and overcoming personal barriers for performing activities of daily living
Performing an activity in which concerns about falls are experienced together with the facilitator^#^
Shifting from self-defeating to self-motivating thoughts regarding activities in which concerns about falls are experienced
Formulating an action plan to repeat the activity performed in this session*

**Session 6: Safe behaviour (telephone; 35 min)**
Review previous session and homework*
Discuss theme of the session using a checklist:
Recognising risk-taking behaviour in daily life Identifying personal risk-taking behaviour in daily life Finding safe alternatives for unsafe behaviour Prioritising fall-risk behaviour and planning behaviour-change strategies
Formulating an action plan to perform safe behaviour*

**Session 7: Managing concerns about falls (telephone; 35 min)**
Review previous session and homework*
Shifting from self-defeating to self-motivating thoughts regarding activities in which concerns about falls are experienced
Finding personal solutions to perform activities safely in which concerns about falls are experienced
Review and evaluation of the programme
Formulating an action plan to perform an activity safely in which concerns about falls are experienced*

*More explicitly addressed in the individual programme compared to the group approach owing to the individual character of the programme and the contracting.

^#^New element in the individual programme.

Note: several intervention elements of the group programme were not included in the in-home programme, among others:

■ practising simple physical exercises

■ explicit addressing of physical risk factors for falls

■ prevention strategies to minimise potential negative consequences of a fall

■ sharing experiences related to concerns about falls and fall prevention

■ a booster session after six months

#### Contents and format of the programme

The in-home programme aims to teach participants how to deal with their concerns about falls and related avoidance of activity, in order to increase their physical, social and functional activities. AMB-Home consists of seven individual sessions, including three home visits (60, 60 and 75 minutes, respectively) and four telephone contacts (35 minutes each). During each session a main theme is addressed. The themes of the programme are: concerns about falls; thoughts about falling; physical exercise; asserting oneself; overcoming personal barriers; safe behaviour; and managing concerns about falls (see Table 1). All sessions have a similar structure: review the previous session (except the first session); discuss the current session's theme; and formulate a concrete and personal action plan, related to the theme discussed. The contents of each session are described in detail in a facilitator's manual, and participants receive printed materials, including background information on the session's theme and worksheets to complete during or between the sessions.

The principles of cognitive restructuring [27] are used for shifting maladaptive to adaptive attitudes with respect to falling, as well as for increasing self-efficacy beliefs and feelings of control. Four strategies are applied to obtain these goals: (1) restructuring misconceptions to promote a realistic view of fall-risk and making concerns about falls controllable; (2) setting realistic goals for increasing activity and safe behaviour; (3) adapting the environment to reduce the fall-risk; and (4) promoting the uptake of daily life activities that are avoided owing to concerns about falls. The following techniques are applied in the programme. Motivational interviewing is used as a discussion technique to encourage the internal motivation and increase the self-efficacy of participants. This technique is a client-centred approach, with reflective listening and positive affirmations rather than direct questioning, persuasion, or advice-giving [28]. In addition, to tailor the programme to the participant's needs and preferences, participants are encouraged to come up with activities that they consider important and which they would like to perform safely. These activities are then incorporated into programme elements such as action planning. Participants experiencing difficulties in recognising such activities are prompted in this process by being shown them 16 drawings of activities of the Iconographical Falls Efficacy Scale (Icon-FES) [29]. Action plans are used to bridge the gap between behavioural intentions and behaviour itself. In every session a personally relevant activity is chosen by the participant. The 'when', 'where', and 'how' to perform the activity, and ways in which to pursue the activity in the face of obstacles, as well as expected challenges and possible solutions are discussed [30]. The activity is supposed to be carried out by the participant prior to the next session. In session 5, a more challenging daily activity (related to concerns about falls) in daily life is first performed under the direct supervision of the facilitator [31]. Furthermore, a DVD is used for modelling by presenting stories of other people reporting concerns about falls, and moreover, to encourage problem-solving skills of the participant, which is one of the core skills of self-management [32]. At last, the participant is encouraged to invite a significant other. This person (often a spouse or other relative, friend or neighbour) is present at the home visits and shown how to help and give support during the programme. An overview of the programme is shown in Table 1.

The AMB-Home programme was facilitated by eight trained nurses, that were qualified in the field of geriatrics and work for home-care agencies. Facilitators received a two-day training in which the manual was studied. During the training, special attention was given to aspects such as motivational interviewing, behavioural change, and 'exposure *in vivo*' to feared activities [31], by professionals in these particular fields. The facilitators were responsible for planning the sessions with the participant, according to the given format and time schedule. Throughout the start of the programme, the researchers periodically observed the facilitators during their contacts with participants. Monthly group meetings of the facilitators and the researchers were held to evaluate and discuss the progress of the trial, the flow of participants, the programme and the performance of the programme by the facilitators.

### Outcomes

#### Effect evaluation

Table 2 presents the outcomes of the effect evaluation.

**Table 2:** Outcome measures of the effect evaluation

Primary outcome measures	Instrument	No. of items	Range*	S	B	FU1	FU2
concerns about falls	FES-I [33,34,53]	16	16 to 64	-	TI	TI	TI
avoidance of activity owing to concerns about falls	FES-IAB	16	16 to 64	-	TI	TI	TI

**Secondary outcome measures**	**Instrument**	**No. of items**	Range*	**S**	**B**	**FU1**	**FU2**

activities of daily life	GARS [35]	18	18 to 72	-	TI	TI	TI
no. of falls	N/A	1	N/A	-	C >	C >	C >
- indoor	N/A	1	N/A	-	C >	C >	C >
- outdoor	N/A	1	N/A	-	C >	C >	C >
no. of times medical attention required as a result of falls	N/A	1	N/A	-	C >	C >	C >

**Tertiary outcome measures**	**Instrument**	**No. of items**	Range*	**S**	**B**	**FU1**	**FU2**

perceived consequences of falling - loss of functional independence subscale	CoF [9]	6	6 to 24	-	TI	TI	TI
perceived consequences of falling - damage to identity subscale	CoF [9]	6	6 to 24	-	TI	TI	TI
catastrophic beliefs about consequences of a fall	CAFS [37]	5	5 to 20	-	TI	TI	TI
perceived control over falling	PCOF [38]	4	4 to 16	-	TI	TI	TI
mastery	Personal Mastery Scale [39]	7	7 to 35	-	TI	TI	TI
feelings of anxiety	HADS-A [40,41]	7	0 to 21	-	TI	TI	TI
symptoms of depression	HADS-D [40,41]	7	0 to 21	-	TI	TI	TI
social support interactions	SSL 12-I [42]	12	12 to 48	-	TI	TI	TI
health-related quality of life	SF-12 [43]	12	12 to 36	-	TI	TI	TI

**Additional variables**	**Instrument**	**No. of items**	Range*	**S**	**B**	**FU1**	**FU2**

demographic data (age, gender, living situation, educational level)	N/A	5	N/A	SQ	-	-	-
perceived general health	Perceived general health [23]	1	1 to 5	SQ	-	-	-
cognitive impairment	AMT4 [24]	4	N/A	-	TI	-	-
	TICS [25]	11	0 to 41	-	TI^#^	-	-
chronic medical conditions	Chronic medical conditions questionnaire [46]	5	0 to 5	-	TI	-	-
concerns about falls	N/A	1	1 to 6	SQ	TI	TI	TI
avoidance of activity owing to concerns about falls	N/A	1	1 to 6	SQ	TI	TI	TI
no. of falls in the previous 6 months	N/A	1	1 to 6	SQ	TI	-	-
no. of falls in the previous 5 months	N/A	1	1 to 6	-	-	TI	-
no. of falls in the previous 7 months	N/A	1	1 to 6	-	-	-	TI
expectations of intervention	Adapted expectations questionnaire [47]	4	4 to 20	-	TI	-	-

*The underlined scores indicate the most favourable scores; N/A = not applicable; S = screening; B = baseline; FU1 = 5-month follow-up; FU2 = 12-month follow-up; SQ = screening questionnaire; TI = telephone interview; C > calendar (continuous registration); ^#^Only assessed if participant fails on the AMT4

#### Primary outcome measures

The primary outcomes of the effect evaluation are concerns about falls and the avoidance of activity as a result of these concerns. Concerns about falls are assessed by the 16-item Falls Efficacy Scale-International (FES-I). Participants are asked to indicate how concerned they are about falling while carrying out several activities of daily living (1 = not all concerned to 4 = very concerned) [33,34]. In addition, when people indicate that they are at least somewhat concerned about falling while carrying out an activity, people are asked to indicate to what extent they avoid the activity as a result of their concerns (FES-IAB; AB indicates Avoidance Behaviour; 1 = never to 4 = often).

#### Secondary outcome measures

Secondary outcomes are disability and the number of falls. Disability is measured by the Groningen Activity Restriction Scale (GARS) [35]. The 18 items of the GARS measure disability in the area of ADL (Activities of Daily Living including mobility) as well as IADL (Instrumental Activities of Daily Living). Participants are asked if they are currently able to perform the activity (1 = yes, fully independently to 4 = no, only with help from others). The number of falls is registered continuously during the course of the trial by a fall calendar. A fall is defined as an event that results in a person coming to rest inadvertently on the ground or on another lower level [36]. If a fall occurs, participants indicate on the calendar: (a) the location of the fall (indoor or outdoor); and (b) the number of times medical attention is received owing to the fall.

#### Tertiary outcome measures

The tertiary outcomes include: the perceived consequences of falling (CoF) with two 6-item subscales ('loss of functional independence' and 'damage to identity') [9]; catastrophic beliefs about the consequences of a fall (CAFS: Catastrophising About Falling Scale, 5 items) [37]; perceived control over falling (PCOF; 4 items) [38]; mastery (7 items) [39]; feelings of anxiety and symptoms of depression with two 7-item subscales of the Hospital Anxiety and Depression Scale (HADS) [40,41]; social support interactions (SSL12-I: Social Support List of Interaction, 12 items) [42]; and health-related quality of life (SF-12: Health Survey, 12 items) [43].

#### Additional variables

Several variables are assessed to provide insight into the population under study, and to interpret the outcomes of the study. The socio-demographic and health-related variables, assessed during the process of screening for eligibility, are: age, gender, living alone or not, educational level, perceived general health (item one of the MOS SF-20) [23,44], and self-reported impaired vision and hearing [45]. Other health-related variables assessed during the baseline measurement are: chronic medical conditions (a 5-item checklist) [46] and cognitive status (AMT4: shortened version of the Abbreviated Mental Test; 4 items and TICS: Telephone Interview Cognitive Status; 11 items) [24,25]. Furthermore, 1-item questions on concerns about falls, the avoidance of activities owing to these concerns and the number of falls are assessed at baseline, as well as at all follow-up measurements. Lastly, at the baseline, participants are asked about their outcome expectations with regard to the programme [47].

### Process evaluation

To determine the feasibility of the programme and to identify factors that may influence its effectiveness, the following outcomes of the process evaluation are assessed: characteristics of the population reached (reach); protocol adherence by facilitators (fidelity); protocol adherence by participants (dose received: exposure); participants and facilitators' opinion about the programme and perceived benefits and achievements (dose received: satisfaction); and experienced barriers and potential solutions for these (barriers) [21,48,49]. Table 3 provides a detailed overview of the outcomes of the process evaluation and their operationalisation during the course of the trial. Data is collected from participants in the programme group, and from the facilitators.

**Table 3:** Outcome measures of the process evaluation

Component and definition	Operationalisation	Measurement						
		SQ	QCf	V	Qf	Qp	If	D
**Reach**								
Proportion of the intended target population that participated in the programme	Characteristics of participants and facilitators	+						+
	Number of participants that refused, dropped out or completed the programme							+
	Reasons for withdrawal							+

**Fidelity**								
Extent to which the programme was implemented as planned	Preparation time and duration of the session		+	+				
	Per session component: extent to which carried out, duration and active participation by the participant		+	+				
	Extent to which the facilitator achieved to:							
	- conveying information to the participant			+	+			
	- having the participant phrase their important activities and how these activities could be performed safely and independently			+	+			
	- having the participant set goals regarding an action plan			+	+			
	- using motivational interviewing techniques			+	+			

**Dose received (exposure)**								
Extent of participants' active engagement in and receptiveness to the programme	Overall opinion of the facilitator/participant regarding the participant's engagement in:							
	- the programme				+	+		
	- the formulation of an action plan and carrying out an action plan				+	+		
	Use of materials		+			+		
	Exposure and adherence to homework		+		+	+		
	Extent to which the participant complied with contracts		+		+	+		
	Quality of action plans formulated by the participants				+	+		

**Dose received (satisfaction)**								
Satisfaction of participants and facilitators with the programme	Overall opinion of the participant					+		
	Experienced benefits, burden, usefulness by the participants				+	+		
	Recommendations to others by participants					+		
	Overall opinion of the facilitator				+		+	

**Barriers**								
The extent to which problems were encountered while applying the programme	Strong and weak aspects of the programme				+	+	+	
	Matters for improvement				+	+	+	

SQ = screening questionnaire filled in by participant before start programme, QCf = questionnaire filled in by facilitator for each participant for each contact, V = voice recording for selection of contacts, Qf = questionnaire filled in by facilitator for each participant after completion of programme, Qp = questionnaire filled in by participant after programme, If = group interview with facilitators, and D = data recorded by researchers during programme period

### Economic evaluation

A cost-effectiveness analysis is carried out in which costs are considered from a societal perspective. The economic evaluation measures and evaluates the 'real' costs. In this study, direct health-care costs are included; i.e. costs incurred by the in-home programme and health-care costs incurred by the participants. The costs of the programme consist of used materials, salaries of the facilitators, costs of training sessions for the facilitators etc. Health-care costs include hospital visits (inpatient and outpatient treatment), GP consultations, visits to paramedics, (nursing) home-care, informal care, and aids and appliances. In order to estimate the costs, the quantity of each resource will be multiplied by its assigned unit cost of price. Cost prices are obtained from the Dutch guidelines for cost analysis in health-care research [50,51]. If such guidelines do not provide for specific health-care use, real costs or tariffs will be used to estimate costs.

### Data collection

Data for the *effect evaluation *is gathered by means of telephone interviews which are conducted by trained interviewers, who are blinded for group allocation. For the assessment of fall accidents, participants received a fall calendar after the baseline measurement. Every month, a sheet of the calendar has to be returned via a freepost envelope. People are reminded by telephone after one-and-half weeks if a sheet is not returned.

The *process evaluation *data is gathered by several means. Participants who complete at least five sessions, fill in a questionnaire to report on the programme's feasibility and usefulness. Facilitators receive a registration form for each participant to report on the time spent per session, the participant's adherence with regard to homework assignments and the extent to which the programme is performed according to protocol. Voice recordings are used in a random selection of the sessions to gather objective data about the performance according to the protocol. Additionally, facilitators fill in questionnaires that assess their opinion about the programme for each participant and their overall opinion of the programme. Researchers conduct short telephone interviews to identify the reason(s) for withdrawal among people who do not complete the programme. Lastly, the researchers conduct a final evaluation meeting with the facilitators to discuss the overall programme.

The fall calendar mentioned before is also used for the collection of the data for the *economic evaluation*. Participants have to report their use of health-care services each month, in addition to their fall accidents.

Non-compliant participants of the programme group are approached for all follow-up measurements, and participants with missing data are contacted to ensure completion of data, as recommended by Hollis and Campbell [52].

Newsletters are sent 4 and 11 months after the baseline measurement, to keep the participants informed about the trial.

### Sample size and power

Sample size calculations are based on outcomes of a previous study using the Falls Efficacy Scale-International (FES-I) among older people in The Netherlands [53]. Two times 112 participants will provide 80% power at alpha 0.05 (one-tailed) to detect differences between the intervention and control groups' mean score of at least 3.8 points (SD is 11.4 equivalent with an effect size of 0.33 on the FES-I). However, a dropout rate of 20% during the study is expected, based on the experiences in the home visit study from Van Haastregt et al. [54]. Therefore, 2 × 140 participants are needed to enrol in the trial.

In a previous Dutch study, 54% of the population reported fear of falling, 38% reported related avoidance behaviour, and 48% reported poor or fair perceived general health [8]. Based on the experiences in that study and on the evaluation of AMB-NL [15], we estimate that approximately 6% of the older people who return the screening questionnaire will meet all inclusion and exclusion criteria and will be interested in participating in the trial. With an estimated response rate of 55%, a minimum sample of 8,200 older persons aged 70 or over needs to be approached with a screening questionnaire.

### Analysis

Descriptive techniques will be used to describe the study groups. Baseline variables will be compared, to detect differences between the participants of both groups at the start of the study. Data of the effect evaluation will be analysed according to both the intention-to-treat and per-protocol principles. In the first analyses all participants will be included according to their original assignment [52]. Participants of the intervention group who attended at least five of the seven programme sessions will be included in the per-protocol analysis. Based on prior work, five sessions of the programme are considered as sufficient programme exposure [15,18]. Mixed-effects regression analyses will be applied, to test for between-group differences with respect to the primary, secondary and tertiary outcome measures at all follow-up assessments. Models will be adjusted for the following covariates, considered as relevant for the outcomes [8]: concerns about falls, age, gender, perceived general health, and number of falls in the past 6 months at baseline. Additional covariates will be included in the analysis if baseline differences are detected for variables relevant for the outcomes. The level of statistical significance will be set at 0.05 (one-tailed). Data on the process and economic evaluation will be analysed and presented using descriptive techniques and appropriate statistical testing.

## Discussion

A multicomponent cognitive behavioural in-home programme has been developed to teach frail older people living in the community how to manage their concerns about falls and related activity avoidance. A trial was conducted to evaluate this programme on effectiveness, feasibility and costs. The screening procedure for eligible participants started in March 2009. For practical reasons, the procedure is distributed across four cycles, that last of which was in December 2009. In the first cycle, 2,250 older people living in the south-east of The Netherlands received the screening questionnaire. Enrolment into the study was disappointing during this cycle. Therefore, we decided to send out more screening questionnaires than planned in the next cycles, and to broaden the inclusion criteria from regular to both some concerns about falls and some avoidance of activity. In addition, responders entered a draw to win one of the fifteen gift vouchers worth € 25. Eventually, 11,490 older people received the screening questionnaire across the four cycles. The response rate was 52.6% and by applying the modified inclusion criteria, 389 participants were included into the study.

Changes made in the treatment protocol of the group programme while adapting it for the individual in-home application may influence effectiveness. First, the physical exercises from the group approach were removed from the programme, because the facilitator could not monitor them adequately during the telephone contacts. Instead, more attention is given to action plans and overcoming a more challenging (fear-related) problem in daily life in session 5. Second, by sending huge numbers of questionnaires, we choose a screening procedure to include enough participants in a relatively short period of time. This is also the reason why participants did not receive a personal assessment in which they are tested on, for example, physical performances, such as balance and strength, and motivation. With this procedure, we may have missed helpful information about how realistic the participants' concerns about falls are and, how much they are prepared to do something about their concerns. In the future, possibilities for testing participants' physical performance [55] and motivation at the start of the programme might be explored [56]. Third, to make implementation in the Dutch health-care setting more acceptable, we have chosen for community nurses as facilitators. Yet, these nurses have little experience of facilitating behavioural change; instead, they are usually trained to provide care and information. In this programme, they were also expected to act as a personal coach to the participants, and to encourage self-management skills. This might have put additional demands on them as facilitators, for which they may not have been trained in the past. Therefore, and to increase the treatment integrity of the programme, nurses received specific training prior to the start of the project. Lastly, the use of telephone contacts as part of the programme is rather new among this older population, in particular, and nurses from home-care organisations in the Netherlands. If successful, the results might lead to a more cost-effective programme and promising prospects for further use of telephone contacts in programmes with frail older people in the future.

### Progress of the study

The baseline measurements started in March 2009. Data on the effect, process and economic evaluation are expected to be available in 2012.

### Future implementation

During the last two years the successful group programme AMB-NL has been implemented nationwide into the Dutch health-care setting. Details about the programme are presented on the Dutch website http://www.zichtopevenwicht.nl. If the results of the current trial show effectiveness and feasibility of AMB-Home, then the in-home programme can be offered to people who are not able or willing to participate in the group programme.

## List of abbreviations

AMB-Home: The Dutch in-home version of A Matter of Balance; AMB-NL: The Dutch group version of A Matter of Balance

## Competing interests

The authors declare that they have no competing interests.

## Authors' contributions

GK and RZ developed the project and obtained funding. All authors participated in the final design of the study. TD is the researcher on the project. TD, RZ and GK developed the materials for the study and received input from the other authors, particularly KD. TD wrote the first draft of this paper and the other authors provided input. All authors read and approved the final manuscript.

## Pre-publication history

The pre-publication history for this paper can be accessed here:

http://www.biomedcentral.com/1472-6963/11/228/prepub
